# Daily hemodialysis practices in Australia/New Zealand and in France: a comparative cohort study

**DOI:** 10.1186/s12882-019-1330-1

**Published:** 2019-05-07

**Authors:** Adélaïde Pladys, Sahar Bayat, Cécile Couchoud, Cécile Vigneau, Stephen McDonald

**Affiliations:** 1 EHESP Rennes, Sorbonne Paris Cité, EA 7449 Reperes, Rennes, France; 2 Renal Epidemiology and Information Network (REIN), Biomedecine Agency, Saint Denis La Plaine, France; 3 University of Rennes 1, INSERM U1085-IRSET, Rennes, France; 4 CHU Pontchaillou, Department of Nephrology, Rennes, France; 5 Australia and New Zealand Dialysis and Transplant Registry (ANZDATA), South Australian Health and Medical Research Institute (SAHMRI), Adelaide, Australia; 6 University of Adelaide, Adelaide, Australia

**Keywords:** ANZDATA registry, Daily hemodialysis, REIN registry, Trajectories, Survival

## Abstract

**Background:**

As patients on daily hemodialysis (DHD) have heterogeneous profiles, DHD benefit in terms of survival is still debated. The aim of this study was to compare DHD practices in France and in Australia and New Zealand*.*

**Methods:**

This study was based on data from the French Renal Epidemiology and Information Network (REIN) and the Australian and New Zealand Dialysis and Transplant Registry (ANZDATA). All incident patients from both registries who underwent DHD (i.e., 5–6 sessions/week, including short daily hemodialysis and long nocturnal hemodialysis) at least once during their trajectories were included, and their characteristics and care trajectories were compared. For survival analyses, one French patient was matched to one Australian or New Zealand patient, based on age, sex and year of dialysis start. Survival was assessed using the Cox proportional hazards model, and access to renal transplantation was evaluated using the Fine & Gray model to take into account death as competing risk.

**Results:**

Between 2003 and 2012, 523 patients from the AZNDATA and 753 from the REIN registry started DHD. ANZDATA patients were younger (54.8 vs 64.0 years, *p* < 0.001) and had comorbidities more frequently than French patients. In both registries, one third of patients were on early DHD (i.e., DHD started less than one year after dialysis initiation). Long nocturnal hemodialysis was more frequent in the ANZDATA than in the REIN cohort (20.8 and 3%, respectively). Comparison of the matched subgroups showed comparable survival rates between French and Australian/New Zealand patients (HRadjusted = 1.08; 95%CI: 0.78–1.50). Access to renal transplantation also was similar between matched groups (SHRadjusted = 1.30, 95%CI: 0.86–1.97).

**Conclusions:**

Our study shows that, despite differences in terms of patients’ characteristics and DHD regimens, the mortality risk and access to renal transplantation are similar in France and Australia and New Zealand.

**Electronic supplementary material:**

The online version of this article (10.1186/s12882-019-1330-1) contains supplementary material, which is available to authorized users.

## Background

Chronic kidney disease (CKD) is a major public health issue with an increasing number of patients treated worldwide [1]. Although hemodialysis (HD) three times per week is the most frequent renal replacement therapy [2], HD regimens have been modified to improve the patients’ quality of life and biological parameters [3–7]. Increasing HD weekly frequency is considered to be the best way to mimic the kidney functional role [8–10]. Several studies have reported that daily HD (DHD) improves hypertension [3, 11, 12] and uremia [4, 8] management in addition to ventricular hypertrophy [8, 12, 13].

In France, the Renal Epidemiological and Information Network (REIN) registry collects data on all patients who start renal replacement therapy in the entire country [14]. Analysis of the REIN data highlighted the clinical feature heterogeneity of patients starting DHD (i.e., 5 or 6 HD sessions/week) [15], and showed that the risk of death is higher in patients on DHD than in matched patients on HD 3 times/week [16]. This confirmed a previous study by Suri et al. that included patients undergoing in-center HD [17]. Conversely, other studies reported that DHD is associated with better survival [18–23]. These contradictory results could be explained by differences in the practices associated with DHD between France and other countries [16, 17].

The Australian and New Zealand Dialysis and Transplant Registry (ANZDATA) has been collecting data on all patients undergoing dialysis and kidney transplantation in Australia and New Zealand for over 40 years. Analysis of ANZDATA data showed that intensive HD is used in both countries, and that various regimens (long nocturnal, short DHD) have been developed mainly for home dialysis [24, 25]. Particularly, long nocturnal DHD and home conventional HD have been implemented since 2001 [26, 27]. Since then, several reviews [25, 26, 28] described the benefits of nocturnal DHD on biological functions and quality of life. A recent study showed (in some analyses) lower mortality with intensive HD, compared with conventional HD [24] in Australia and New Zealand.

As the REIN and ANZDATA registries collect similar data, we decided to compare the characteristics, care trajectories, survival and access to renal transplantation of French and Australian/New Zealand patients undergoing DHD to highlight possible differences.

## Methods

### Population

The REIN registry was established in 2002, and since 2011 covers the entire French territory. The ANZDATA registry started to collect data on renal dialysis and kidney transplantation in Australia and New Zealand in 1977. REIN and ANZDATA include all patients treated by renal replacement therapy (dialysis or kidney transplantation). After inclusion, REIN collects data annually and when dialysis modalities change, while ANZDATA only collects data annually. The organization of the two registries has been described in detail elsewhere [14, 29].

This study was a retrospective analysis of prospectively collected REIN and ANZDATA data on all incident patients aged 18 years and over who were treated at least once with DHD during their care trajectories in France or in Australia/New Zealand between January 1, 2003 and December 31, 2012 (inclusion period). The study endpoint was set at December 31, 2013 to have a minimum of one-year follow-up for the patients included in the study. DHD was defined as 5 or 6 HD sessions/week (short daily or long nocturnal HD). The ANZDATA data did not allow following patients from DHD initiation. Patients were described and followed from the first date of renal replacement therapy registered in the database until death, transplantation, or the study endpoint (December 31, 2013).

### Data collection

Three categories of variables were included: i) patients’ demographic and clinical/laboratory data at DHD initiation: sex, age, hemoglobin rate, body mass index (BMI), smoking status (current, former and never smoker), and comorbidities, such as diabetes, peripheral vascular disease (PVD), cerebrovascular disease (CVD), coronary disease and respiratory insufficiency; ii) DHD modalities: conventional DHD (at least 5 sessions/week) or convective DHD (hemofiltration, hemodiafiltration and biofiltration), number of weekly sessions and duration of each session; iii) patients’ care trajectories: date and dialysis modalities at first registration in the database (at dialysis start in REIN, and at the first annual survey in ANZDATA), and clinical outcome at the end of the follow-up: death, kidney transplantation, or endpoint.

### Statistical analyses

#### Descriptive analyses

Continuous variables were described as median and interquartile range (IQR), or were grouped in clinically relevant classes. Categorical variables were described as frequencies and percentages. The sub-groups’ characteristics (French vs Australian/New Zealand patients) were compared with the Chi square test.

#### Matching procedure

To evaluate and compare survival and access to renal transplantation between countries, patients with homogenous DHD modalities in terms of session duration were selected. Consequently, all patients on long nocturnal HD (5–6 sessions/week; ≥5 h/session) were excluded, because this modality is rare in France. Patients on short daily HD (5–6 sessions/week; < 5 h/session) from the ANZDATA registry were randomly matched 1:1 with patients from the REIN registry, based on age (±1 year), sex and year of dialysis initiation.

#### Outcome analyses

The first primary outcome was patient survival. Patients were followed from the first date of dialysis recorded in the database until death, or were censored at renal transplantation or at the endpoint (December 31, 2013). The second primary outcome was access to renal transplantation. For this, patients were followed until transplantation, death, or the endpoint. Cox regression analysis was used to evaluate the association between patients’ characteristics and death and renal transplantation in the matched population (the matching structure was taken into account in the survival analyses). Death occurrence during the follow-up was considered as a competing event for renal transplantation. To take into account this competing risk, a Fine & Gray model was applied, and renal transplantation was considered as a time-dependent covariate in survival analyses. Kaplan-Meier survival curves and Cumulative Incidence Functions were plotted for each group. All variables associated with the event of interest in the univariate model (*p*-value < 0.2) were included in the multivariate model. The 95% Confidence Interval (CI) was calculated for all Hazard Ratio (HR) and Subdistribution Hazard Ratio (SHR), and a p-value < 0.05 was considered as statistically significant.

Statistical analyses were performed with the Stata 13.1 software (College station, TX).

## Results

### Patients’ characteristics at DHD initiation

Between 2003 and 2012, 453 patients in Australia, 70 in New Zealand, and 753 in France started DHD. During this period, 59,438 new patients started HD in the REIN registry, and 20,133 in ANZDATA (16,960 in Australia and 3173 in New Zealand).

The median age at DHD initiation was 55.5 years (IQR: 45.7–66.2) in Australia, 51.2 (IQR: 41.3–61.9) in New Zealand, and 64.0 years (IQR: 50.7–76.3) in France. Patients from New-Zealand were younger and more obese than patients from Australia; however, comorbidity rates were comparable (see Additional file 1: Table S1 for the details of this comparison). As the number of patients from New Zealand was low and they displayed characteristics similar to those of the Australian patients, patients from these two countries were grouped in a single Australia/New Zealand cohort.

The percentage of patients with low hemoglobin concentration (< 10 g/dl) was higher in the French than in the Australian/New Zealand cohort (31.5% vs 18.2%, *p* < 0.001; Table 1). Conversely, the percentage of men (70% vs 63.5%) and the median BMI (29 kg/m^2^ vs 25.3 kg/m^2^) were higher in the Australian/New Zealand than in the French cohort. Moreover, Australian/New Zealand patients had more comorbidities than French patients, particularly PVD (30.6% vs 25.2%, p < 0.001) and coronary disease (42.3% vs 26.4%, p < 0.001).

**Table 1:** Characteristics at DHD initiation of patients included in the ANZDATA and REIN registries

	ANZDATA *n* = 523	REIN *n* = 753	
	n (%)	n (%)	p
Socio-demographic data			
Sex			0.02
Men	366 (70)	478 (63.5)	
Women	157 (30)	275 (36.5)	
Age at DHD start			< 0.001
Median (IQR)	54.8 (44.7–65.0)	64.0 (50.7–76.2)	
Bio-clinical data			
Tobacco			0.01
No-smoker	208 (39.8)	366 (48.6)	
Current	75 (14.3)	100 (13/3)	
Former	237 (45.3)	205 (27.2)	
Missing	3 (0.6)	82 (10.9)	
Hemoglobin (g/dl)			0.01
< 10	95 (18.2)	237 (31.5)	
10–12	242 (46.3)	276 (36.7)	
> 12	178 (34.0)	191 (25.4)	
Missing	8 (1.5)	49 (6.5)	
BMI (kg/m2)			0.01
< 18.5	9 (1.7)	50 (6.6)	
18.5–23	82 (15.7)	158 (21)	
23–25	50 (9.6)	105 (13.9)	
25–30	147 (28)	171 (22.7)	
≥ 30	229 (44)	159 (21.2)	
Missing	6 (1)	110 (14.6)	
Diabetes			0.08
Yes	212 (40.5)	291 (38.6)	
No	311 (59.5)	455 (60.4)	
Missing	0 (0.0)	7 (0.0)	
PVD			0.01
Yes	106 (30.6)	190 (25.2)	
No	363 (69.4)	545 (72.4)	
Missing	0 (0.0)	18 (2.4)	
CVD			0.01
Yes	74 (14)	75 (10.0)	
No	449 (86)	595 (79.0)	
Missing	0 (0.0)	83 (11.0)	
Coronary disease			0.01
Yes	221 (42.3)	199 (26.4)	
No	302 (57.7)	539 (71.6)	
Missing	0 (0.0)	15 (2.0)	
Respiratory insufficiency			0.01
Yes	90 (17.2)	116 (15.4)	
No	433 (82.8)	620 (82.3)	
Missing	0 (0.0)	17 (2.3)	
DHD features			
DHD frequency per week			< 0.001
Median (IQR)	5 (5–6)	6 (5–6)	
DHD hours per session			< 0.001
Median (IQR)	3 (3–4)	3 (2–3)	
DHD environment			< 0.001
At home	6 (1.1)	43 (5.8)	
In-center	470 (89.9)	547 (72.6)	
Satellite unit	47 (9)	163 (21.6)	

*DHD* Daily hemodialysis, *BMI* Body Mass Index, *PVD* Peripheral vascular disease, *CVD* Cerebrovascular disease

### Patients’ care trajectories

**Fig. 1:**
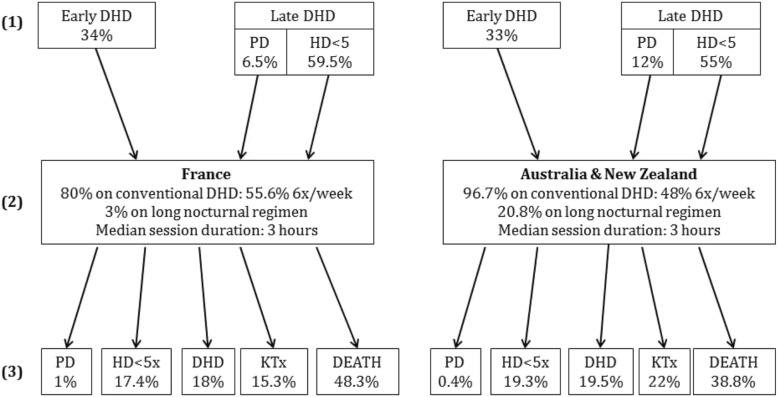
Dialysis modalities at first registration in the registry (1), DHD modalities, (2) and last medical status (3) by country: France (left panels) and Australia/New Zealand (right panels). DHD: daily hemodialysis; Early DHD: placement on DHD less than 1 year after dialysis initiation; Late DHD: placement on DHD more than 1 year after dialysis initiation; PD: peritoneal dialysis; HD: hemodialysis; KTx: Kidney transplantation

Placement on early DHD was defined as starting DHD less than one year after renal replacement therapy initiation, and placement on late DHD was defined as starting DHD more than one year after inclusion in the registry. In France as well as in Australia and New Zealand, one third of patients were on early DHD, and the others on late DHD (Fig. 1). The more common modality was HD < 5 times/week in all countries, whereas PD was more frequently used by Australian and New Zealand patients than French patients (12% vs 6.5%). In the Australia/New Zealand cohort, patients on early and on late DHD presented similar characteristics. Conversely, French patients on early DHD were older, with low hemoglobin concentration (< 10 g/dl), and fewer comorbidities than those on late DHD (for more details, see Additional file 1: Table S2).

In France, 80% of patients used conventional DHD and 20% convective DHD (hemofiltration, hemodiafiltration or biofiltration), whereas in Australia and New Zealand, patients used almost exclusively conventional DHD (96.7%). The median DHD session duration was 3 h, whatever the country. Long nocturnal DHD (5 or 6 sessions/week with ≥5 h per session) was more common in Australia and New Zealand than in France (20.8% vs 3%).

At the end of the follow-up, among the patients still on dialysis, the proportions of patients on DHD, HD or PD were comparable between countries (Fig. 1). Death rate (per 100 people, per year) was 3.5 and 4.4 in Australia/New Zealand and in France, respectively. Concomitantly, more patients underwent kidney transplantation in the Australian/New Zealand than in the French cohort (22% vs 15.3%).

### Patients’ outcomes (matched patients on DHD)

For the survival analyses, patients on nocturnal DHD were excluded (*n* = 131). The matching procedure (age, sex, year of dialysis start) allowed matching 226 patients from the French cohort with 226 patients of the Australia/New Zealand cohort. Their mean age was 56.3 ± 14.7 years and the male to female ratio 2.77. After matching, the percentage of patients with comorbidities remained higher in the Australia/New Zealand than in the French cohort (for more details, see Additional file 1: Table S3).

**Fig. 2:**
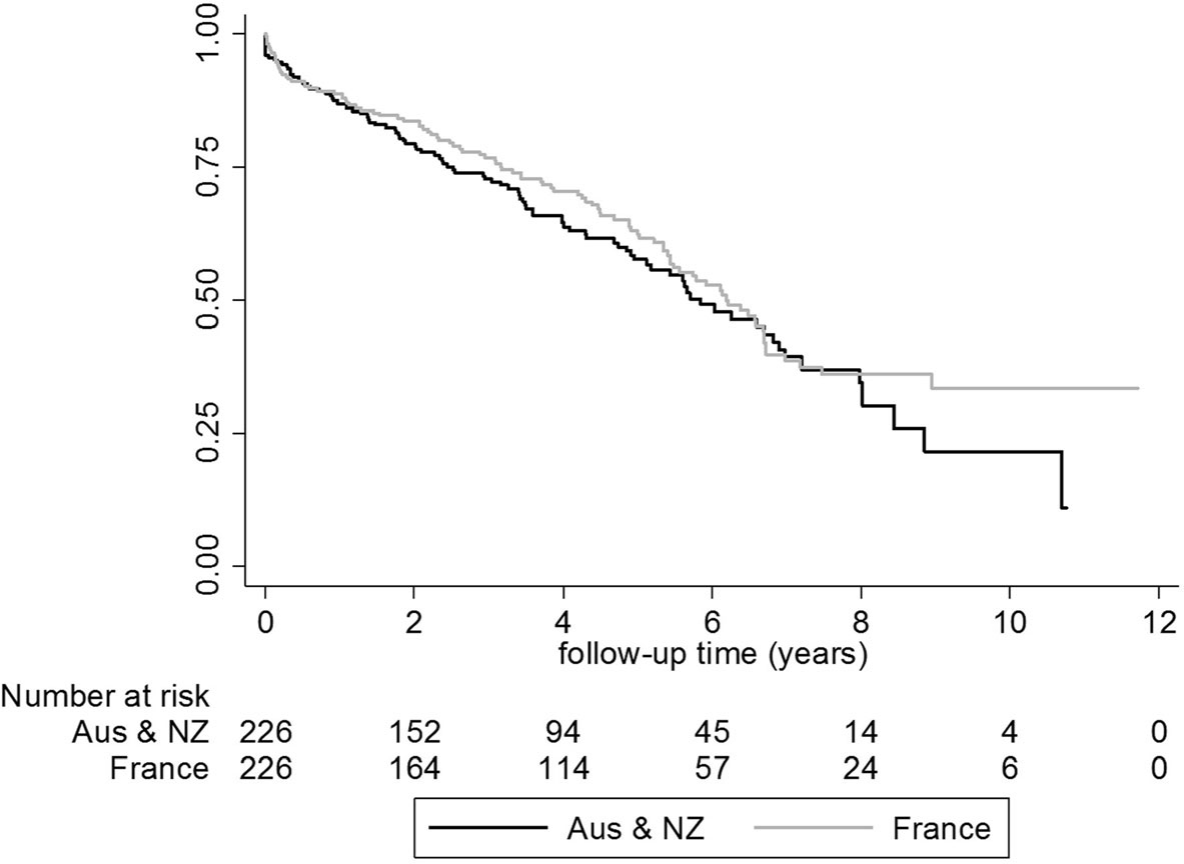
Crude Kaplan Meier survival curves for matched patients on DHD by country: Australia/New Zealand (black line) and France (gray line)

By the end of 2013, 106/226 (46.9%) French patients and 101/226 (44.7%) Australian/New Zealand patients were dead. The survival of matched patients from inclusion in the database to death is represented in crude Kaplan Meier mortality curves (Fig. 2). These curves overlapped during the follow-up. In the adjusted model that included clinical/laboratory data, country was not an independent factor associated with the mortality risk (HRadjusted = 1.08; 95%CI: 0.78–1.50; Table 2).

**Table 2:** Unadjusted and adjusted specific Hazard Ratio (HR) for the risk of death in the matched cohort

	Unadjusted HR (95% CI)	Adjusted HR (95% CI)
Smoking status (vs no smoker)		
Current/former smoker	1.49 (1.12–1.99)	–
Missing	1.16 (0.58–2.32)	–
Hemoglobin (vs 10–12 g/dl)		
< 10	1.25 (0.89–1.76)	1.13 (0.79–1.61)
> 12	0.60 (0.43–0.84)	0.72 (0.51–1.01)
Missing	0.59 (0.26–1.34)	0.46 (0.20–1.10)
BMI (vs 18.5–23 kg/m^2^)		
< 18.5	1.56 (0.83–2.94)	1.56 (0.81–2.99)
23–25	1.03 (0.63–1.69)	0.82 (0.49–1.37)
25–30	1.07 (0.71–1.60)	0.99 (0.65–1.53)
≥ 30	0.79 (0.51–1.21)	0.62 (0.39–0.99)
Missing	1.79 (1.02–3.16)	2.01 (1.12–3.62)
Diabetes (vs no)		
Yes	1.86 (1.41–2.44)	1.42 (1.03–1.96)
PVD (vs no)		
Yes	2.53 (1.92–3.32)	1.87 (1.34–2.63)
CVD (vs no)		
Yes	2.52 (1.81–3.51)	1.56 (1.09–2.25)
Missing	2.56 (1.63–4.03)	3.87 (2.34–6.39)
Coronary disease (vs no)		
Yes	2.49 (1.89–3.28)	1.87 (1.33–2.64)
Respiratory disease (vs no)		
Yes	1.61 (1.18–2.20)	–
Country (vs France)		
Australia & New Zealand	1.07 (0.81–1.40)	1.08 (0.78–1.50)
Renal graft during the follow-up (vs no)		
Yes	0.21 (0.1–0.45)	0.35 (0.16–0.76)
Late DHD		
Early DHD (placed on DHD less than 1 year after dialysis initiation)	1.92 (1.44–2.56)	2.43 (1.76–3.35)
Session duration (hours)	0.82 (0.67–1.0)	–

*BMI* Body Mass Index, *PVD* Peripheral vascular disease, *CVD* Cerebrovascular disease, *HR* Hazard Ratio, *CI* Confidence Interval

**Fig. 3:**
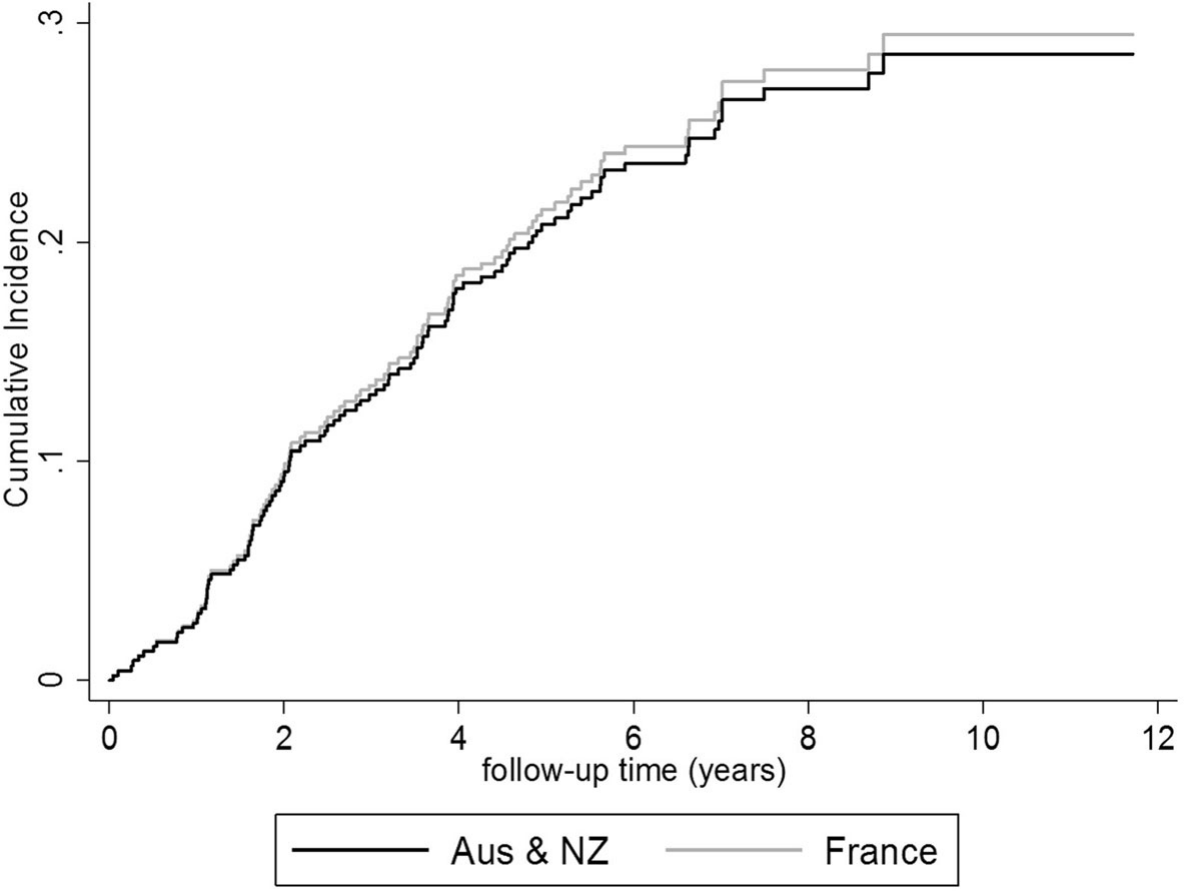
Crude cumulative incidence function for access to renal transplantation of matched patients on DHD by country: Australia/New Zealand (black line) and France (gray line)

During the follow-up, 57/226 (25.2%) French patients and 51/226 (22.6%) Australian/New Zealand patients underwent kidney transplantation. Their demographic and clinical profiles were comparable (for more details, see Additional file 1: Table S4), and their crude cumulative incidence function for access to renal transplantation overlapped during the analysis time (Fig. 3). The probability to have access to renal transplantation was comparable in the two matched groups (censored at death; HRadjusted = 1.36, 95%CI: 0.91–2.05; these results are described with more details in Additional file 1: Table S5).

## Discussion

This is the first study that describes and compares the characteristics and estimated survival rates of all incident patients treated at least once with DHD during their care trajectories in Australia/New Zealand and France between 2003 and 2012.

France, Australia and New Zealand are very different countries with specific lifestyles, healthcare systems, and disease/comorbidity prevalence. France is a democratic republic with centralized, universal health coverage: the national health insurance system covers the entire population. Australia and New Zealand also have national health insurance schemes that provide universal coverage, including access to dialysis treatment. Australia has a federal government where the healthcare system is divided across levels of government. In all three countries, population is ageing. As a consequence, prevalence of chronic diseases, such as CKD, is increasing. In 2013, the overall incidence of renal replacement therapy was 160 per million population (pmp), 110 pmp, and 123 pmp in France [30], Australia, and New Zealand [31], respectively. The REIN and ANZDATA registries have been established in these countries to monitor the incidence, prevalence and outcomes associated with end-stage renal disease (ESRD). Although the REIN registry is much younger than the ANZDATA registry, they collect similar types of data and have the same objectives. Therefore, their data could be used to study and compare DHD practices in Australia/New Zealand and France.

This study shows that patients who started DHD in Australia and New Zealand between 2003 and 2012 were younger, with high BMI, and mainly cardiovascular comorbidities. Conversely, in France, they were older, with less comorbidities but died rapidly. Moreover, in all three countries, DHD modalities were not widely established during the study time. Indeed, the percentage of incident patients on DHD during the study period was very low (~ 1% in France and ~ 2% in Australia/New Zealand). Differently from the United States and Canada where DHD has been used for a long time [3, 4, 21], in France, knowledge about DHD practices was poor before 2016. In Australia and New Zealand, Marshall et al., evaluated frequent/extended dialysis modalities, but did not specifically study short DHD [24, 32]. The costs and constraints of in-center DHD are higher than those of PD or HD 3 times/week [3, 33]. These facts could limit in-center DHD prescription by nephrologists and its acceptance by patients, and could also explain why DHD was not much implemented in France, Australia and New Zealand during the study period. However, the new low-flow DHD modality at home is slowly progressing in France since 2012, and practices associated with frequent HD might change in the future, at least in France [33, 34].

Despite the infrequent DHD use in all three countries, we observed several country-specific differences in terms of DHD practices. In our previous studies on French patients, we reported the heterogeneity of clinical features and care trajectories of patients on DHD (15,16). In agreement, in the present French cohort, patients on early DHD were very different from patients on late DHD. Overall, French patients on early DHD were older and with several comorbidities, although we previously identified also a subgroup of young patients with high access to renal transplantation [16]. Therefore, in France, DHD is mainly used by two groups: 1) older and frail patients, presumably in response to cardiac instability and comorbidities, and 2) young patients to maintain their quality of life before kidney transplantation [15, 16]. Conversely, in the Australia/New Zealand cohort, patients on early DHD and late DHD were comparable. Overall, Australian/New Zealand patients were younger, with more comorbidities than the French ones. Nevertheless, a similar distinction between old and young subgroups of patients on DHD could be made also in Australia and New Zealand.

Besides the patients’ profile differences, we also observed differences in care trajectories for patients on DHD. First, among Australian/New Zealand patients on late DHD, a high proportion had PD as first dialysis modality (12% vs 6.5% in France). PD was very common in Australia and New Zealand before 2000 (more than 30% of dialyzed patients on PD, mainly at home) [27, 35]. After 2000, long nocturnal HD and home HD have been progressively put in place [27, 35]. Accordingly, 20.8 and 3% of patients on DHD in Australia/New Zealand and in France, respectively, underwent long nocturnal DHD. In France, home HD and nocturnal HD were very rare until 2012, probably because of the many care facilities (in center, satellite units) that can cover the patients’ demand in the entire country [36] and the smaller home-facility distance compared with Australia and New Zealand.

On the other hand, the care trajectories after DHD initiation were comparable in the three countries. Among patients still alive at the endpoint, a similar proportion of patients in Australia/New Zealand and in France were still on dialysis (HD < 5 sessions/week or DHD; Fig. 1).

Despite differences in clinical features (less comorbidities in the French group), the mortality risk and access to kidney transplantation were comparable in Australia/New Zealand and France in the matched population. The characteristics of patients who underwent kidney transplantation also were comparable, suggesting similar selection criteria in these three countries. We hypothesized that survival rate might be higher among Australian/New Zealand patients than among French patients with ESRD on dialysis. Based on ANZDATA annual report (2013), the mortality rate per 100 patient-years was 13.1 (95%CI: 12.5–13.8) for dialysis-dependent patients in Australia and 13.7 (12.3–15.2) in New Zealand [37]. In France, the one-year survival was 83.2% (95%CI: 82.9–83.5) for the 2002–2013 incident patients [38]. We could not compare survival of patients with ESRD in the three countries because in the national annual reports, for survival analyses, transplanted patients were censored in the ANZDATA [37], but not in the REIN registry [38]. Survival rate might not be the best way to highlight the benefits associated with DHD, thus explaining the contradictory results of previous studies [16–23]. Alternatively, the analysis of the patients’ quality of life could help, but this information is not routinely collected in registries.

The major strength of our study is that this is the first comparison of DHD associated practices in France and Australia/New Zealand. Furthermore, thanks to the REIN and ANZDATA registries, we could include a large population-based cohort on DHD between 2003 and 2012 and we could take into account various clinical characteristics.

Our study has several limitations. The medical reasons explaining the nephrologist’s decisions to start or to switch to DHD were not recorded in the REIN and ANZDATA registries, raising the possibility of selection bias. Differently from the REIN registry where data are collected at renal replacement therapy initiation, at every dialysis modality change, at death, at renal transplantation and also annually, ANZDATA collects data only annually. Therefore, the dates of dialysis initiation, DHD initiation, death and renal transplantation are registered in ANZDATA at the survey update, and the follow-up times calculated for the Australian/New-Zealand cohort were less consistent. The study design allowed us to analyze the global survival of patients who started DHD between 2003 and 2012 in Australia/New Zealand and France, but not the survival specifically for the period they underwent DHD. Indeed, we could not identify the exact duration of DHD treatment for each included patient. Finally, we could study only the data collected in both registries; for example, we did not have any information on the patients’ income or quality of life.

## Conclusions

Our study shows that practices associated with DHD are different in France and Australia/New Zealand, possibly due to geographical factors. Additional studies on DHD indications are needed to complete our observations and understand why age- and sex-matched patients from the two cohorts presented comparable survival despite their clinical differences.

## Additional file

Additional file 1: **Table S1.** Comparison of the characteristics at DHD initiation of incident patients from Australia and New Zealand”. This table contains the description and comparison of the characteristics of patients on DHD from Australia and New Zealand. **Table S2.** Characteristics of incident patients from the ANZDATA (Australia and New Zealand) and REIN (France) registry according to the starting dialysis modality”. This table compares the characteristics of patients according to their starting dialysis modality per registry: left panel, patients from the ANZDATA registry, and right panel, patients from the REIN registry. **Table S3.** Characteristics of age- and sex-matched patients by country. To compare access to renal transplantation and survival, one French patient was matched (sex, age and year of dialysis start) to one patient from Australia or New Zealand. This table compares the matched patients’ characteristics. **Table S4.** Characteristics of matched patients who underwent renal transplantation by country. This table summarizes the characteristics of patients who underwent renal transplantation among the matched patients. **Table S5.** Unadjusted and adjusted specific Hazard Ratios (HR) and Subdistribution Hazard Ratios (SHR) for renal transplantation. This table contains the results of the univariate and multivariate Cox (left panel) and Fine & Gray regressions (right panel) for the event of interest (access to renal transplantation). (DOCX 41 kb)

## Abbreviations

ANZDATA: Australian and New Zealand Dialysis and Transplant Registry
BMI: Body Mass Index
CI: Confidence Interval
CKD: Chronic Kidney Disease
CVD: Cerebrovascular disease
ESRD: End Stage Renal Disease
HD: Hemodialysis
HR: Hazard Ratio
IQR: Interquartile range
PD: Peritoneal Disease
PVD: Peripheral vascular disease
REIN: Renal Epidemiological and Information Network
SHR: Subdistribution Hazard Ratio

## References

[1] Liyanage T, Ninomiya T, Jha V (2015). Worldwide access to treatment for end stage kidney disease: a systematic review. Lancet..

[2] Cambi V, Savazzi G, Arisi L, Bignardi L, Bruschi G, Rossi E, Migone L (1975). Short Dialysis schedules (SDS)- finally ready to become a routine?. Proc Eur Dial Transplant Ass.

[3] Blagg CR, Ing TS, Berry D, Kjellstrand CM (2004). The history and rationale of daily and nightly hemodialysis. Contrib Nephrol.

[4] Kjellstrand CM, Ing T. Daily hemodialysis, history and revival of a superior method. ASAIO J. 1998:117–22. 9617939

[5] Kooistra M (2003). Frequent prolonged home haemodialysis: three old concepts, one modern solution. Nephrol Dial Transplant.

[6] Punal J, Lema LV, Sanhez-Guisande D, Ruano-Ravina A (2008). Clinical effectiveness and quality of life of conventional haemodialysis versus short daily haemodialysis: a systematic review. Nephrol Dial Transplant.

[7] Vos PF, Zilch O, Kooistra MP (2001). Clinical outcomes of daily Dialysis. Am J Kidney Dis.

[8] Bonomini V, Mioli V, Albertazzi A, Scolari P (1998). Daily dialysis programme: indications and results. Nephrol Dial Transplant.

[9] Kjellstrand CM, Evans RL, Petersen RJ, Shideman JR, Von Hartitzsch B, Buselmeier TJ (2004). The "unphysiology" of dialysis: a major cause of dialysis side effects?. Hemodial Int.

[10] Toussaint ND (2010). Review: differences in prescription between conventional and alternative haemodialysis. Nephrol..

[11] Culleton B, Asola MR (2011). The impact of short daily and nocturnal hemodialysis on quality of life, cardiovascular risk and survival. J Nephrol.

[12] Locatelli F, Buoncristiani U, Canaud B, Köhler H, Petitclerc T, Zucchelli P (2005). Dialysis dose and frequency. Nephrol Dial Transpl.

[13] Lindsay R, Nesrallah G, Suri R, Garg A, Moist L (2004). Is more frequent hemodialysis benefit and what is the evidence?. Curr Opin Nephrol Hypertens.

[14] Couchoud C, Stengel B, Landais P (2006). The renal epidemiology and information network (REIN): a new registry for end stage renal disease in France. Nephrol Dial Transplant.

[15] Pladys A, Bayat S, Kolko A, Béchade C, Couchoud C, Vigneau C (2016). On the behalf of the REIN registry. French patients on daily hemodialysis: clinical characteristics and treatment trajectories. BMC Nephrol.

[16] Pladys Adélaïde, Vigneau Cécile, Hourmant Maryvonne, Duneau Gabrielle, Couchoud Cécile, Bayat Sahar (2018). Association between daily haemodialysis, access to renal transplantation and patients' survival in France. Nephrology.

[17] Suri R, Lindsay RM, Bieber BA, Pisoni RL, Garg AX, Austin PC, Moist LM, Robinson BM, Gillepsie BW, Couchoud CG, Galland R, Lacson EK, Zimmerman DL, Li Y (2013). Nesrallah GE for the IQDR. A multinational cohort study of in-center daily hemodialysis and patient survival. Kidney Int.

[18] Johansen KL, Zhang R, Huang Y, Chen SC, Blagg CR, Goldfarb-Rumyantzev AS, Hoy CD, Lockridge RS, Miller BW, Eggers PW, Kutner NG (2009). Survival and hospitalization among patients using nocturnal and short daily compared to conventional hemodialysis: a USRDS study. Kidney Int.

[19] Kjellstrand CM, Buoncristiani U, Ting G, Traeger J, Piccoli GB, Sibai-Galland R, Young BA, Blagg CR (2008). Short daily haemodialysis: surival in 415 patients treated for 1006 patient-years. Nephrol Dial Transplant.

[20] Kjellstrand C, Buoncristiani U, Ting G, Traeger J, Piccoli GB, Siabai-Galland R, Young BA, Blagg CR (2010). Survival with short-daily hemodialysis: association of time, site, and dose of dialysis. Hemodial Int.

[21] Weinhandl ED, Liu J, Gilberston DT, Arneson TJ, Collins AJ (2012). Survival in daily home hemodialysis and matched thrice-weekly in-center hemodialysis patients. J Am Soc Nephrol.

[22] Nesrallah GE, Lindsay RM, Cuerden MS, Garg AX, Port F, Austin PC, Moist LM, Pierratos A, Chan CT, Zimmerman D, Lockridge RS, Couchoud C, Chazot C, Ofsthun N, Levin A, Copland M, Courtney M, Steele A, McFarlane PA, Geary DF, Pauly RP, Komenda P, Suri R (2012). Intensive hemodialysis associates with improved survival compared with conventional hemodialysis. J Am Soc Nephrol.

[23] Woods J, Port FK, Orzol S, Buoncristiani U, Young E, Wolfe RA, Held PJ (1999). Clinical and biochemical correlates of starting "daily" hemodialysis. Kidney Int.

[24] Marshall MR, Polkinghorne KR, Kerr PG, Hawley CM, Agar JWM, McDonald SP (2016). Intensive hemodialysis and mortality risk in Australian and New Zealand populations. Am J Kidney Dis.

[25] JWM A (2005). Nocturnal Haemodialysis in Australia and New Zealand. Nephrology..

[26] Agar JWM, Somerville CA, Dwyer KM, Simmonds RE, Boddington JM, Waldron CM (2003). Nocturnal hemodialysis in Australia. Hemodial Int.

[27] Agar JWM, Hawley CM, Kerr PG (2011). Home hemodialysis in Australia and New Zealand: how and why it has been successful. Semin Dial.

[28] Kerr PG, Agar JWM, Hawley CM (2011). Alternate night nocturnal hemodialysis: the Australian experience. Semin Dial.

[29] McDonald SP, Russ GR (2013). Australian registries—ANZDATA and ANZOD. Transplant Rev (Orlando).

[30] REIN annual report 2013. 2013 ESRD incidence rates 2013;39–84. Available at: https://www.agence-biomedecine.fr/IMG/pdf/rapport_rein2013.pdf

[31] ANZDATA Registry. 37th Report, Chapter 1: incidence of end stage kidney disease. Australia and New Zealand Dialysis and transplant registry, Adelaide, Australia. 2015. Available at: http://www.anzdata.org.au/anzdata/AnzdataReport/38thReport/c01_anzdata_incidence_v1.0_20160108_web.pdf

[32] Marshall MR, Hawley CM, Kerr PG, Polkinghorne KR, Marshall RJ, Agar JWM, McDonald SP (2011). Home hemodialysis and mortality risk in Australian and New Zealand populations. Am J Kidney Dis.

[33] Lee CP, Zenios SA, Chertrow GM (2008). Cost-effectiveness of frequent in-center hemodialysis. J Am Soc Nephrol.

[34] Kohn O, Coe F, Ing T (2010). Solute kinetics with short-daily home hemodialysis using slow dialysate flow rate. Hemodial Int.

[35] Agar JWM (2010). Review: Understanding sorbent dialysis systems. Nephrology..

[36] Bayat S, Macher MA, Couchoud C, Bayer F, Lassale M, Villar E, Caillé Y, Mercier C, Joyeux V, Noel C, Kessler M, Jacquelinet C (2015). Individual and regional factors of access to the renal transplant waiting list in France in a cohort of dialyzed patients. Am J of Transplant.

[37] ANZDATA Registry. 36th Report, Chapter 3: deaths. Australia and New Zealand Dialysis and transplant registry, Adelaide; 2014. Available at: http://www.anzdata.org.au/anzdata/AnzdataReport/36thReport/2013c03_deaths_v1.4.pdf.

[38] Chantrel F., de Cornelissen F., Deloumeaux J., Lange C., Lassalle M. (2013). Survie et mortalité des patients en IRCT. Néphrologie & Thérapeutique.

