# Supplementary material for: Daily hemodialysis practices in Australia/New Zealand and in France: a comparative cohort study
Source: BMC Nephrol. 2019 May 7;20:156. doi: 10.1186/s12882-019-1330-1 (PMC6505110; doi:10.1186/s12882-019-1330-1)
Supplement: Supplementary file 1 — Table S1. Comparison of the characteristics at DHD initiation of incident patients from Australia and New Zealand”. This table contains the description and comparison of the characteristics of patients on DHD from Australia and New Zealand. Table S2. Characteristics of incident patients from the ANZDATA (Australia and New Zealand) and REIN (France) registry according to the starting dialysis modality”. This table compares the characteristics of patients according to their starting dialysis modality per registry: left panel, patients from the ANZDATA registry, and right panel, patients from the REIN registry. Table S3. Characteristics of age- and sex-matched patients by country. To compare access to renal transplantation and survival, one French patient was matched (sex, age and year of dialysis start) to one patient from Australia or New Zealand. This table compares the matched patients’ characteristics. Table S4. Characteristics of matched patients who underwent renal transplantation by country. This table summarizes the characteristics of patients who underwent renal transplantation among the matched patients. Table S5. Unadjusted and adjusted specific Hazard Ratios (HR) and Subdistribution Hazard Ratios (SHR) for renal transplantation. This table contains the results of the univariate and multivariate Cox (left panel) and Fine & Gray regressions (right panel) for the event of interest (access to renal transplantation). (DOCX 41 kb) [file 12882_2019_1330_MOESM1_ESM.docx]

**Additional file 1**

**Table S1. Comparison of characteristics at DHD initiation of incident patients from Australia and New Zealand**

|  | **Asutralia**  **N=453** | **New Zealand**  **N=70** |  |
| --- | --- | --- | --- |
|  | **n (%)** | **n (%)** | **p** |
| **Sex** |  |  | 0.782 |
| Men | 318 (70.2) | 48 (68.6) |  |
| Women | 135 (29.8) | 22 (31.4) |  |
| **Age at DHD start** |  |  | 0.0068 |
| Mean ± std | 55.6 ± 15.6 | 50.3 ± 13.4 |  |
| **Tobacco** |  |  | 0.510 |
| No-smoker | 175 (38.6) | 33 (47.1) |  |
| Current | 67 (14.8) | 8 (11.4) |  |
| Former | 208 (46.0) | 29 (45.5) |  |
| Missing | 3 (0.6) | 0 (0.0) |  |
| **Hemoglobin (g/dl)** |  |  | 0.0273 |
| Mean ± std | 11.5 ± 1.6 | 11.0 ± 1.4 |  |
| **BMI at DHD start (kg/m2)** |  |  | 0.0037 |
| Mean ± std | 30.2 ± 8.0 | 33.3 ± 10.3 |  |
| **Diabetes** |  |  | 0.719 |
| Yes | 185 (40.8) | 27 (38.6) |  |
| No | 268 (59.2) | 43 (61.4) |  |
| **PVD** |  |  | 0.131 |
| Yes | 144 (31.8) | 16 (23) |  |
| No | 309 (68.2) | 54 (77) |  |
| **CVD** |  |  | 0.483 |
| Yes | 66 (14.6) | 8 (11.4) |  |
| No | 387 (85.4) | 62 (88.6) |  |
| **Coronary disease** |  |  | 0.234 |
| Yes | 196 (43.3) | 25 (35.7) |  |
| No | 257 (56.7) | 45 (64.3) |  |
| **Respiratory insufficiency** |  |  | 0.988 |
| Yes | 78 (17.2) | 12 (17) |  |
| No | 375 (82.8) | 58 (83) |  |

BMI: Body Mass Index; CVD: Cerebrovascular disease; PVD: Peripheral vascular disease.

**Table S2. Characteristics of incident patients from the ANZDATA (Australia and New Zealand) and REIN (France) registry according to the starting dialysis modality**

|  | **Australia and New Zealand** | | | **France** | | |
| --- | --- | --- | --- | --- | --- | --- |
|  | **Late DHD** | **Early DHD** |  | **Late DHD** | **Early DHD** |  |
|  | **n=350** | **n=173** |  | **n=496** | **n=257** |  |
|  | n (%) | n (%) | p | n (%) | n (%) | p |
| **Sex** |  |  | 0.85 |  |  | 0.02 |
| Men | 244 (69.7) | 122 (70.5) |  | 330 (66.5) | 148 (57.6) |  |
| Women | 106 (30.3) | 51 (29.5) |  | 166 (33.5) | 109 (42.4) |  |
| **Age at DHD start (years)** |  |  | 0.21 |  |  | 0.01 |
| 18-45 | 86 (24.6) | 45 (26.0) |  | 103 (20.8) | 42 (16.3) |  |
| 45-60 | 145 (41.4) | 62 (35.8) |  | 129 (26) | 44 (17.2) |  |
| 60-75 | 88 (25) | 41 (23.7) |  | 144 (29) | 72 (28) |  |
| ≥75 | 31 (9) | 25 (14.5) |  | 120 (24.2) | 99 (38.5) |  |
| **Tobacco** |  |  | 0.51 |  |  | 0.01 |
| No-smoker | 134 (38.3) | 74 (42.8) |  | 232 (46.8) | 134 (52) |  |
| Current | 51 (14.6) | 24 (13.9) |  | 73 (14.7) | 24 (9.3) |  |
| Former | 162 (46.3) | 75 (43.4) |  | 147 (29.6) | 58 (22.6) |  |
| Missing | 3 (0.8) | 0 (0.0) |  | 44 (8.9) | 38 (14.8) |  |
| **Hemoglobin (g/dl)** |  |  | 0.04 |  |  | 0.01 |
| <10 | 59 (17) | 36 (20.8) |  | 114 (23.0) | 123 (48) |  |
| 10-12 | 170 (48.6) | 72 (41.6) |  | 209 (42) | 67 (26) |  |
| >12 | 119 (34) | 59 (34.1) |  | 152 (30.6) | 39 (15.2) |  |
| Missing | 2 (0.6) | 6 (3.5) |  | 21 (4.2) | 28 (10.8) |  |
| **BMI (kg/m2)** |  |  | 0.23 |  |  | 0.01 |
| <18.5 | 4 (1.1) | 5 (2.9) |  | 27 (5.4) | 23 (9) |  |
| 18.5-23 | 50 (14.3) | 32 (18.5) |  | 106 (21.4) | 52 (20.2) |  |
| 23-25 | 32 (9.1) | 18 (10.4) |  | 70 (14) | 35 (13.6) |  |
| 25-30 | 95 (27.1) | 52 (30.1) |  | 132 (26.6) | 39 (15.2) |  |
| ≥30 | 164 (47) | 65 (37.6) |  | 117 (23.6) | 42 (16.3) |  |
| Missing | 5 (1.4) | 1 (0.6) |  | 44 (9) | 66 (25.7) |  |
| **Diabetes** |  |  | 0.18 |  |  | 0.01 |
| Yes | 149 (42.6) | 63 (36.4) |  | 194 (39) | 97 (37.7) |  |
| No | 201 (57.4) | 110 (63.6) |  | 302 (61) | 153 (59.5) |  |
| Missing | 0 (0.0) | 0 (0.0) |  | 0 (0.0) | 7 (2.7) |  |
| **PVD** |  |  | 0.32 |  |  | 0.01 |
| Yes | 112 (32.0) | 48 (27.7) |  | 134 (27) | 56 (21.8) |  |
| No | 238 (68.0) | 125 (72.3) |  | 358 (72.2) | 187 (72.8) |  |
| Missing | 0 (0.0) | 0 (0.0) |  | 4 (0.8) | 14 (5.4) |  |
| **CVD** |  |  | 0.69 |  |  | 0.01 |
| Yes | 51 (14.6) | 23 (13.3) |  | 50 (10) | 25 (9.7) |  |
| No | 299 (85.4) | 150 (86.7) |  | 413 (83.3) | 182 (70.8) |  |
| Missing | 0 (0.0) | 0 (0.0) |  | 33 (6.7) | 50 (19.5) |  |
| **Coronary disease** |  |  | 0.04 |  |  | 0.01 |
| Yes | 159 (45.4) | 62 (35.8) |  | 134 (27.0) | 65 (25.3) |  |
| No | 191 (54.6) | 111 (64.2) |  | 360 (72.6) | 179 (69.6) |  |
| Missing | 0 (0.0) | 0 (0.0) |  | 2 (0.4) | 13 (5.1) |  |
| **Respiratory insufficiency** |  |  | 0.35 |  |  | 0.01 |
| Yes | 64 (18.3) | 26 (15.0) |  | 81 (16.4) | 35 (13.6) |  |
| No | 286 (81.7) | 147 (85.0) |  | 412 (83) | 208 (80.9) |  |
| Missing | 0 (0.0) | 0 (0.0) |  | 3 (0.6) | 14 (5.4) |  |

Early DHD: being placed on DHD less than one year after renal replacement therapy initiation; Late DHD: being placed on DHD more than one year after being registered in the registry. BMI: Body Mass Index; CVD: Cerebrovascular disease; PVD: Peripheral vascular disease.

**Table S3. Characteristics of age- and sex-matched patients by country***

|  | **Australia/ New Zealand**  **N=226** | **France**  **N=226** |  |
| --- | --- | --- | --- |
|  | **n (%)** | **n (%)** | **p** |
| **Sex** |  |  | 0.91 |
| Men | 167 (74) | 168 (74.3) |  |
| Women | 59 (26) | 58 (25.7) |  |
| **Age at DHD start (years)** |  |  | 0.99 |
| 18-45 | 37 (16.4) | 37 (16.4) |  |
| 45-60 | 88 (39) | 86 (38) |  |
| 60-75 | 62 (27.4) | 65 (28.8) |  |
| ≥75 | 39 (17.3) | 38 (16.8) |  |
| **Tobacco** |  |  | 0.01 |
| No-smoker | 86 (38) | 109 (48.2) |  |
| Current | 30 (13.3) | 33 (14.5) |  |
| Former | 110 (48.7) | 63 (28) |  |
| Missing | 0 (0.0) | 21 (9.3) |  |
| **Hemoglobin (g/dl)** |  |  | 0.01 |
| <10 | 38 (16.8) | 57 (25.2) |  |
| 10-12 | 107 (47.3) | 80 (35.4) |  |
| >12 | 78 (34.5) | 74 (32.7) |  |
| Missing | 3 (1.3) | 15 (6.6) |  |
| **BMI (kg/m2)** |  |  | 0.01 |
| <18.5 | 6 (2.7) | 16 (7) |  |
| 18.5-23 | 32 (14.2) | 47 (20.8) |  |
| 23-25 | 23 (10.2) | 35 (15.5) |  |
| 25-30 | 71 (31.4) | 60 (26.5) |  |
| ≥30 | 93 (41.2) | 38 (16.8) |  |
| Missing | 1 (0.4) | 30 (13.3) |  |
| **Diabetes** |  |  | 0.04 |
| Yes | 102 (45) | 80 (35.4) |  |
| No | 124 (55) | 146 (64.6) |  |
| **PVD** |  |  | 0.01 |
| Yes | 84 (37.2) | 59 (26) |  |
| No | 142 (62.8) | 167 (74) |  |
| **CVD** |  |  | 0.01 |
| Yes | 45 (20) | 23 (10.2) |  |
| No | 171 (75.7) | 174 (77) |  |
| Missing | 0 (0.0) | 29 (12.8) |  |
| **Coronary disease** |  |  | 0.01 |
| Yes | 116 (51.3) | 57 (25.2) |  |
| No | 110 (48.7) | 169 (74.8) |  |
| **Respiratory insufficiency** |  |  | 0.18 |
| Yes | 47 (20.8) | 36 (16) |  |
| No | 179 (79.2) | 190 (84) |  |

BMI: Body Mass Index; PVD: Peripheral vascular disease; CVD: Cerebrovascular disease

*Matching procedure 1:1 age (±1 year), sex and year of dialysis initiation

**Table S4. Characteristics of matched patients who underwent renal transplantation by country***

|  | **Australia/ New Zealand**  **N=51** | **France**  **N=57** |  |
| --- | --- | --- | --- |
|  | **n (%)** | **n (%)** | **p** |
| **Sex** |  |  | 0.76 |
| Men | 38 (74.5) | 41 (72) |  |
| Women | 13 (25.5) | 16 (28) |  |
| **Age at DHD start** |  |  | 0.86 |
| 18-45 | 19 (37.3) | 22 (38.5) |  |
| 45-60 | 28 (55) | 29 (51) |  |
| 60-75 | 4 (7.7) | 6 (10.5) |  |
| **Tobacco** |  |  | 0.04 |
| No-smoker | 25 (49) | 30 (52.6) |  |
| Current | 6 (11.8) | 10 (17.6) |  |
| Former | 20 (39.2) | 12 (21) |  |
| Missing | 0 (0.0) | 5 (8.8) |  |
| **Hemoglobin (g/dl)** |  |  | 0.19 |
| <10 | 3 (6.0) | 7 (12.3) |  |
| 10-12 | 20 (39.2) | 23 (40.4) |  |
| >12 | 28 (54.8) | 24 (42) |  |
| Missing | 0 (0.0) | 5 (8.7) |  |
| **BMI (kg/m2)** |  |  | 0.08 |
| <18.5 | 3 (6.0) | 2 (3.5) |  |
| 18.5-23 | 7 (13.7) | 8 (14.0) |  |
| 23-25 | 7 (13.7) | 12 (21) |  |
| 25-30 | 16 (31.4) | 21 (36.8) |  |
| ≥30 | 18 (35.3) | 9 (15.8) |  |
| Missing | 0 (0.0) | 5 (8.8) |  |
| **Diabetes** |  |  | 0.25 |
| Yes | 8 (15.7) | 14 (24.6) |  |
| No | 43 (84.3) | 43 (75.4) |  |
| **PVD** |  |  | 0.85 |
| Yes | 5 (9.8) | 5 (8.8) |  |
| No | 46 (90.2) | 52 (91.2) |  |
| **CVD** |  |  | 0.12 |
| Yes | 5 (9.8) | 2 (3.5) |  |
| No | 46 (90.2) | 52 (91.2) |  |
| Missing | 0 (0.0) | 3 (5.3) |  |
| **Coronary disease** |  |  | 0.93 |
| Yes | 6 (11.8) | 7 (12.3) |  |
| No | 45 (88.2) | 50 (87.7) |  |
| **Respiratory insufficiency** |  |  | 0.33 |
| Yes | 4 (7.8) | 2 (3.5) |  |
| No | 47 (92.2) | 55 (96.5) |  |

BMI: Body Mass Index; PVD: Peripheral vascular disease; CVD: Cerebrovascular disease

*Matching procedure 1:1 age (±1 year), sex and year of dialysis initiation

**Table S5. Unadjusted and adjusted specific Hazard Ratios (HR) and Subdistribution Hazard Ratios (SHR) for renal transplantation**

|  | **Specific Cox model** | |
| --- | --- | --- |
|  | **Unadjusted HR**  **(95% CI)** | **Adjusted HR**  **(95% CI)*** |
| **Smoking status (*vs* no smoker)** |  |  |
| Current/former smoker | 0.78 (0.53-1.14) | - |
| Missing | 0.77 (0.31-1.91) | - |
| **Hemoglobin (*vs* 10-12)** |  |  |
| <10 | 0.54 (0.27-1.08) | - |
| >12 | 1.32 (0.88-1.98) | - |
| Missing | 0.62 (0.19-2.0) | - |
| **BMI (*vs* 18.5-23)** |  |  |
| <18.5 | 1.65 (0.60-4.55) | 1.54 (0.56-4.24) |
| 23-25 | 1.89 (0.96-3.72) | 2.25 (1.14-4.44) |
| 25-30 | 1.67 (0.92-3.72) | 1.78 (0.96-3.27) |
| ≥30 | 1.01 (0.54-1.90) | 1.01 (0.53-1.95) |
| Missing | 1.24 (0.72-1.54) | 1.09 (0.39-3.04) |
| **Diabetes (*vs* no)** |  |  |
| Yes | 0.41 (0.25-0.65) | - |
| **PVD (*vs* no)** |  |  |
| Yes | 0.25 (0.13-0.43) | 0.38 (0.19-0.74) |
| **CVD (*vs* no)** |  |  |
| Yes | 0.49 (0.23-1.04) | - |
| Missing | 0.46 (0.15-1.46) | - |
| **Coronary disease (*vs* no)** |  |  |
| Yes | 0.24 (0.13-0.43) | 0.35 (0.19-0.66) |
| **Respiratory disease (*vs* no)** |  |  |
| Yes | 0.26 (0.11-0.59) | 0.41 (0.18-0.96) |
| **Country (*vs* France)** |  |  |
| Australia and New Zealand | 1.06 (0.72-1.54) | 1.36 (0.91-2.05) |
| **Late DHD** |  |  |
| Early DHD | 1.43 (0.94-2.18) | - |
| **Session duration (hours)** | 1.0 (0.77-1.31) | - |

BMI: Body Mass Index; PVD: Peripheral vascular disease; CVD: Cerebrovascular disease
